# Forecasting Maternal Complications Based on the Impact of Gross National Income Using Various Models for Rwanda

**DOI:** 10.1155/2020/7692428

**Published:** 2020-08-19

**Authors:** Jean Pierre Namahoro, Adrien Mugabushaka

**Affiliations:** ^1^Department of Mathematics and Physics, China University of Geosciences (Wuhan), Wuhan, China; ^2^School of Mathematics and Physics, College of Science and Technology, Kigali, Rwanda; ^3^School of Applied Sciences, Rwanda Polytechnic (IPRC) University, Kigali, Rwanda

## Abstract

**Introduction:**

Preferably maternal mortalities are predominant in low- and middle-income countries (LMICs). In some African countries, including Rwanda, programs related to health-care delivery to reduce significantly severe complications including mortalities are established. Unfortunately, historical and forecasted maternal mortality reduction and the influence of gross national income (GNI) were not accessed. This study is aimed to forecast the three years of maternal mortalities (MMs) based on the influence of gross national income (GNI) in Rwanda.

**Methods:**

The period involved is from January 2009 to April 2018. Data analyzed were obtained from the Central Hospital of the University of Kigali (CHUK) and mined data from the WHO database. Time series approach (Box-Jenkins and exponential smoothing) and linear regression models were applied. Besides, IBM-SPSS and Eviews were used in the analysis.

**Results:**

The results revealed that MMs were not statistically different in several years, and there was a significant correlation between MMs and GNI (-0.610, *P* value 0.012 < 0.05). A double exponential smoothing model (DESM) was fitted for the best forecast and ARIMA (0,1,0) and linear regression models for a quick forecast.

**Conclusion:**

There was a slight effect of GNI in maternal mortality reduction, which leads to the steady decrease of the forecasted maternal mortality up to May 2021. The Government of Rwanda should intensively strengthen the health-care system, save the children programs, and support pregnant women by using GNI for reducing MMs at an advanced level.

## 1. Introduction

Maternal mortalities (MMs) are unacceptably issued with a higher rate in low- and middle-income countries (LMICs) [1]. Most of those mortalities occur during labor, delivery, or the first 24 hours postpartum, and most complications are hard to be predicted. Timely diagnosis and suitable intervention are not consistent with the prevalence of pregnant women, whereby about 1/350 global pregnancies lead to high maternal mortality risks due to several causes, including hemorrhage, infection, unsafe abortion, eclampsia, and obstructed labor [2–4]. In LMICs, the dying probability of pregnancy women from related causes is closer to 1/50, and it is also rising MMs [5]. Pregnant women are encouraged to adopt health-care services and counseling and get financial support from governments to avoid complications during giving birth [6].

The social economic and environmental factors such as literacy status income and cultural factors persist in LMCs to cause high maternal mortality [7]. As a result, there is a bit of difference in child mortality between mothers. Those who have higher education and wealth status reported a lower rate of child mortality compared to those with less advantageous socioeconomic problems [8]. Although some countries in sub-Saharan Africa have ineffective health policies, several countries established programs for satisfying mothers. Some of these programs are the free maternal services in the implementation of the free maternal health-care system (Kenya) [9], the decision-making of women on their health autonomy of women and place of delivery (Ghana) [10], utilization of maternal health-care services and identifying affecting factors (Ethiopia) [11], and maternal deaths correlated with infectious diseases prevention (Uganda) [12]. Gross national income (GNI) is apparently involved in all those programs.

In Rwanda, the impact of GNI to reduce MMs has not yet scientifically identified. The only suggestive causes of the soaring those mortalities are visible. Those causes are irregular prenatal consultations for pregnant women and insufficient supports during pregnancy and birth processes, and the number of pregnant women sometimes is greater than the midwives [13]. Additionally, the fact of infrastructure (roads) in rural villages and carrying pregnant women to referral-level care are, therefore, crucial. Furthermore, unforeseen factors disturb the needed and feasible interventions [14]. In the greatest effort to reduce maternal mortalities, Rwanda put the related policy ranked 5^th^ among eight Millennium Development Goals (MDGs) [15]. Unfortunately, the highest gross national budget share is used in the programs that fuel their further agenda. In contrast, programs aimed at improving the living standard of the people are only visible on papers [16, 17], and maternal mortalities were not particularly forecasted.

Various models, including Box-Jenkins and exponential smoothing, are time series approaches and, together with the linear regression model (LRM), were used for analyzing and forecasting time-varying events [18]. Due to their simplicity, effective results, and being preferable to a few observations, they have been recently applied to examine and forecast maternal mortality [19–23]. In this study, however, these approaches used to forecast maternal mortality based on the impact of GNI in Rwanda. The initiated objectives are comparing maternal mortality within previous years, showing the potential impact of GNI on the mortality reduction, and forecasting the monthly maternal mortality in three years.

## 2. Methods

In this study, we used the secondary longitudinal monthly maternal mortalities data provided by CHUK, yearly child mortality, and gross national income recorded from 2002 mined in World Health Organization (WHO) database. Wilcoxon Rank Sum Test was used to compare maternal mortality within previous years. The two popular categories of a model of time series approach (Box-Jenkins and exponential smoothing) together with linear regression models were used to forecast maternal mortality for three years. Pearson's correlation was used to show the relationship between GNI and yearly maternal mortality. IBM-SPSS version 2, and Eviews version 9 were used to analyze the data. Here, there is a brief introduction to the models used in this study.

### 2.1. Box-Jenkins Model

Box-Jenkins model consists of ARIMA model written as ARIMA (*p*, *d*, *q*) where AR (*p*) is the autoregressive model with the number of nonseasonal differencing autoregressive term (*p*), and MA (*q*) is the moving average model with some nonseasonal moving differencing (*q*), and the integrated part with *d* as differencing order (*I*). ARIMA model contains seasonal part written as SARIMA (*p*, *d*, *q*) *X* (*P*, *D*, *Q*)s where these upper letters represent the seasonal part, *s* is the number of periods per season, *P* is the number of seasonal autoregressive (SAR) terms, *D* is the number of seasonal differences, and *Q* is the number of the seasonal moving averages (SMAs) [24, 25].

If the series is seasonal with *s* periods per year, then a seasonal ARIMA (SARIMA) model can be written as

(1) $$\begin{array}{c} \Phi_{p}\left(B^{s}\right)\Phi_{p}\left(B\right){\left(1-B\right)}^{d}.{\left(1-B^{s}\right)}^{D}\left(Y_{t}-\mu \right)=\theta_{q}\left(B\right).\Theta_{Q}\left(B^{s}\right)\varepsilon_{t}, \end{array}$$

where

(2) $$\begin{array}{c} \Phi_{p}\left(B^{S}\right)=1-\Phi_{1}.B^{s}-\Phi_{2}B^{2s}-\ldots -\Phi_{p}B^{ps}, \end{array}$$

with

(3) $$\begin{array}{c} \Phi_{p}\left(B\right)=1-\Phi_{1}B-\Phi_{2}B^{2}-\ldots -\Phi_{P}B^{p} \end{array}$$

for Φ_*p*_ ≠ 0 and *θ*_*q*_(*B*)=1 − *θB* − *θ*_2_*B*^2^ − …−*θ*_*q*_*B*^*q*^.

Θ_*Q*_(*B*^*s*^)=1 − Θ*B*^*s*^ − Θ_2_*B*^2*S*^ − …−Θ_*Q*_*B*^*QS*^ for Θ_*q*_(*B*) ≠ 0, where Φ and Θ denote the polynomials *B*^*s*^ of *P* and *Q*, respectively. The most useful polynomial model for seasonal data is the SARIMA model of order (0; 1; 1)X (0; 1; 1) *s* for monthly data *s* = 12.

This model can be given as

(4) $$\begin{array}{c} \left(1-B\right)\left(1-B^{12}\right)=\left(1+\theta \left(B\right)\right)\left(1+\theta \left(B^{2}\right)\right). \end{array}$$

### 2.2. Exponential Smoothing Model

When the model is not the ARIMA, then it can be an exponential smoothing model (ESM). By plotting time-varying data and because the series has neither trends nor seasonal components, the model becomes simple exponential smoothing (SESM). The SES model is written as *S*_*t*_=*σ∗Y*_*t*_+(1 − *σ*)*∗S*_*t*−1_ where *S*_t_ is a smoothed value for time *t*, *a* is a smoothing constant rise between 0 and 1, and *t* = 1, 2, 3; for more details, see [26]. In the case, time-varying data contains a simple trend, and SESM becomes a double exponential smoothing model (DESM) and is used to solve the simple trends associated with two equations applied to estimate the parameter as follows:

(5) $$\begin{array}{c} S_{t}=\alpha Y_{t}+\left(1-\alpha \right)\cdot \left(S_{t\mathrm{-1}}+b_{t\mathrm{-1}}\right)\cdot 0\leq \alpha <1, \end{array}$$

(6) $$\begin{array}{c} b_{t}=ϒ\left(S_{t}-S_{t\mathrm{-1}}\right)+\left(1-ϒ\right)b_{t\mathrm{-1}}\;0\leq ϒ<1, \end{array}$$

where *b*_*t*_is the auxiliary smoothed value which is over time *t* in DESM and *ϓ* is a smoothing constant rising between 0 and 1. Based on the setting initial values to smoothed and auxiliary smoothed values, three suggestions for the starting value of b_1_ can be obtained, *b*_1_ = *y*_2_-*y*_1_, *b*_1_ = 1/3 [(*y*_2_-*y*_1_) + (*y*_3_ - *y*_2_) + (*y*_4_ - *y*_3_)], and *b*_1_ = (*y*_*n*_ − *y*_1_)/*n* − 1 . The first smoothing equation adjusts*s*_*t*_ directly for the trend of the previous period*b*_*t*−1_, by adding it to the last smoothed value*s*_*t*−1_. These computations help to eliminate the lag and bring *s*_*t*_to the appropriate base of the current value. The second smoothing equation then updates the trend, which is expressed as the difference between the last two values. The values for *a* and *?* can be obtained via nonlinear optimization techniques that can be seen in [27, 28].

### 2.3. Linear Regression Model

The linear regression model (LRM) is one of the simplest generalized linear models that can be learned in parameters or variables and/or even both. In this model, it is assumed that the response and explanatory variables are significantly correlated, and the least square estimation approach is well known to be used for the estimation of its parameters; for details, see [29]. In this study, maternal mortality and time are the response and explanatory variables, respectively.

### 2.4. Model Selection Criteria

To identify an appropriate time series model for using, several criteria were followed. In this case, we plotted time-varying data, checked the stationarity of the data, applied transformation if it is necessary, and found the most significant autocorrelation at any lags. The last step is the model checking by following these substeps: plot the multiple plots of original and fitted observations and check if fitted series follow the behavior of original data, are close together, capture the variations within the original data, and then plot the residuals against time [24].

### 2.5. Wilcoxon Rank Sum Test

This test is a nonparametric test used to analyze nonnormal distributed data. In this study, Bera-Jarque estimate used to confirm the abnormality of the mortality series by dividing them into two independent populations [29]. Population 1 is 2009–2013 with *n*_*1*_ to be the past mortalities, and population 2 is 2014–2018 with *n*_2_ to the early mortalities. Then, by Wilcoxon Rank Sum Test, populations were ranked as follows: *n*_1_ + *n*_2_ measurements from 1 (smallest) to *n*_1_ + *n*_2_ (largest) and then adjusting for ties by averaging the ranks that the measurements have received. We then compute *T*_1_, the rank sum for measurements from the past mortalities 1, and *T*_2_, the rank measurements from the early mortalities 2 as detailed in [30]. The difference between these two groups was tested through the following hypothesis (two tails):

- H_**0**_ : there is no true median difference in the level of monthly maternal mortality.
- H_**1**_ :  there is the true median difference in the level of monthly maternal mortality.
- Mathematically, these hypotheses can be written as follows: H0 : *M*1 – *M*2 = 0 and H1 : *M*1 – *M*2 ≠ 0, where M1 and M2 are the medians of past mortalities and early mortalities, respectively. The test statistics is minimum (*T*1, *T*2); then, if *T* ≤ *T*_*O*_ (*T*_*O*_ is from the table at the *a*-*a* level of significance), we will reject the null hypothesis (*H*0).

## 3. Results

### 3.1. Fit the Model and Forecast Maternal Mortality

111 observations were analyzed, modeled, interpreted, and forecasted in the next three years (2018 to 2021). Firstly, monthly maternal mortality series were plotted without transformation, to identify whether the trend of series is signiﬁcant, and then stationary conditions of the series have checked and determined whether there are seasonal variations in the series.

**Figure 1:**
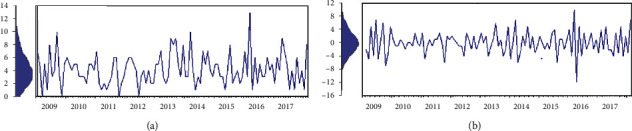
The maternal mortality series (original and transformed). (a) Original series. (b) Transformed (1^st^ difference).

From Figure 1, the series is observed to be an additive model and nonstationary. It presents a partial trend, nonstationary in mean and variance, and seasonal variations. Differencing (1) technique was used to transform the series to be stationary and removing the regular pattern, trends, and seasonal changes. Through ACF and PACF, there is a significant peak at the first lag, which simply looks like the ARIMA model. After transformation to induce stationarity in mean and variance, the stationarity is then not statistically significant. Consequently, the Box-Jenkins approach is doubted to be considered; afterward, the analysis showed that there were two possible models, Box-Jenkins ARIMA (0, 1, 0) random walk model and DESM. The next step is to fit those two models and check their significance and decide the best-fit model to predict future maternal mortality.

**Figure 2:**
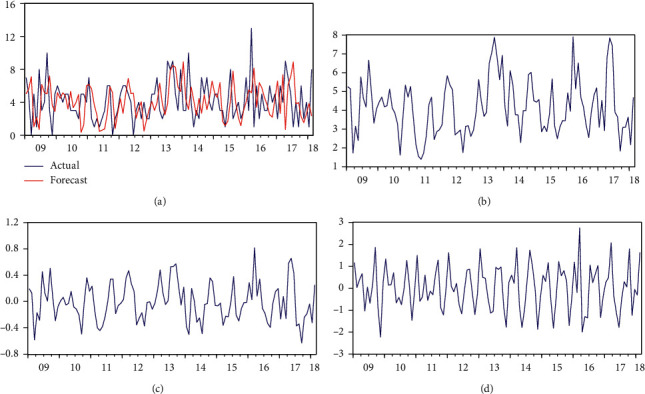
The trajectories of actual and monthly maternal mortality, level, trends, and the season started from 2009 to 2018. (a) Actual and forecast. (b) Level. (c) Trend. (d) Season.

Figure 2 represents multiple plots of original and fitted observations of maternal mortality, which are overlapping, closer, and moving together in the same direction. The series contains regular trends, seasonality, and variable (high to low) higher and lower level mortalities. Since the fitted values follow the behavior of the original series and capture all variations within the original series, the time series approaches statistically fit the data. Even though the parameter estimates of DSEM are not statistically significant (*P* value >0.05) and standard deviations are not too small enough, ARIMA model estimates are all statistically significant (*P* value <0.05), and they can show all variations in the maternal mortality. In addition, the linear regression model is slightly good (R-square = 0.56, *P* value >0.05) (Table 1).

### 3.2. The ACF and PACF in Monthly and Yearly Maternal Mortalities and GNI

In monthly and annual maternal mortalities data from 2009 to 2018, the *P* value of the Ljung-Box Q statistics of each lagged month of four months was less than 0.05. The absolute values of the autocorrelation (ACF) coefficients showed strong associations during the first two lagged months and annually in the first lagged month of GNI for partial autocorrelations (PAC). These outputs show the significant relationship of GNI to reduce mortalities. The computations of model parameters and validation criteria and correlations between GNI and maternal mortality are detailed in an additional file.

### 3.3. Evaluating Monthly Maternal Mortality in Different Periods

Bera–Jarque procedures indicate that Skewness and Kurtosis do not hold the conditions of normality. The data is skewed on the right side because of skewness = 1.195 and Kurtosis = 2.324. Now, the Wilcoxon rank test was used to test whether the medians of the two groups are different, group 1: January 2009 to April 2014 and group 2: May 2014 to April 2018. The null hypothesis fails to be rejected since the test statistic *Z* = -1.299^*∗*^, and it is in the accepted region. Therefore, there is no significant difference in the monthly maternal mortality in the past and early mortalities (Table 1).

### 3.4. Models Comparison

Based on the model checking procedures, DESM in (i) of Table 2 is the best-fitted model to forecast monthly maternal mortality, though it is complicated for applying. The random walk ARIMA (0, 1, 0) model in (ii) of Table 2 and fitted linear regression model (iii) of Table 2 is simpler to forecast the maternal mortality than the DESM model. On the other hand, DESM and a linear regression model produce closer results (Table 2).

### 3.5. Forecasted Maternal Mortality

Table 3 represents the monthly forecast of maternal mortality from May 2018 to August 2018 by using different models and the yearly forecasted maternal mortalities by the use of DESM in three years (2018–2021). In contrast, the total mortality will be 147 with monthly confident intervals (0, 10). In the fact that the actual value of maternal mortality (52) and predicted value (54) from May 2017 to May 2018 are closer, the fitted model is adequate for maternal mortality in CHUK. This reason cannot ignore results obtained by using ARIMA and linear regression model since they are easier to be applied and provide a reliable forecast than DESM.

### 3.6. Impact of GNI in Reducing Maternal Mortality

**Figure 3:**
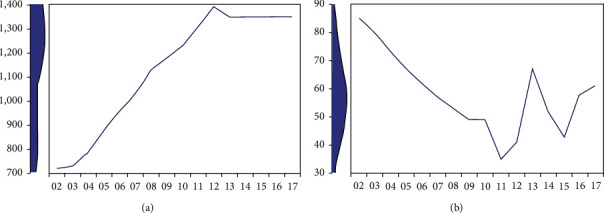
The effect of GNI in yearly maternal mortality reduction from 2002 to 2017. (a) Gross national income. (b) Maternal motility.

The Pearson correlation between yearly maternal mortalities and GNI is statistically significant (*P* value<0.05), which is moving in the opposite direction, meaning that as GNI increases, maternal mortality decreases. The increment rate of GNI from 2002 to 2011 corresponds to the decrement rate of maternal mortality. Even though there was an increase in mortality from 2012 to 2014, GNI seems constant in that time (Table 2 and Figure 3).

## 4. Discussion

This study compared maternal mortality of previous years and forecasted the next three years of the same mortality based on the influence of gross national income (GNI) by using DESM, linear regression, and ARIMA models. The 111 observations have been used for monthly maternal mortality series recorded from January 2009 to April 2018, together with yearly maternal mortality and GNI from 2002 to 2017.

The model identification revealed that the data showed indistinguishable partial trends and nonstationarity, and ACF and PACF, spikes at all four lags, were representing the same information. By applying differencing (1), all partial trend has removed. This result indicated that the monthly maternal mortality series contained trends, and DESM and ARIMA (0, 1, 0) (random drift) models are appropriate. Again, referring to the model identification, model checking, and model fitting for time series approaches, both models are approximately similar to fit the monthly maternal mortality. Because the application of DESM in the forecast seems complicated and not friendly with users, the ARIMA model is mostly applied instead. For instance, it was used in maternal mortalities prediction in Ghana [19] and Norway [31], and the results were similar to those obtained in this study.

**Figure 4:**
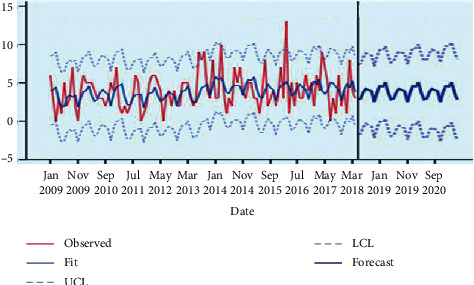
Forecasted monthly maternal mortality from May 2018 to May 2021.

The forecasted value of maternal mortality using DESM will steadily decrease up to May 2021, where the total monthly maternal mortality will be equal to 147 with confident limits of (0, 10) (Figure 4 and Table 3). These results are similar to those established by Maral DerSarkissian in African countries, where he showed that the mean maternal mortality ratios decreased from 695.82 in 1990 to 562.18 in 2005 and achieved MDG-5 pillar [32]. On the other hand, there was no significant difference in the maternal mortalities of previous years. Furthermore, a negative correlation between yearly mortality and GNI indicated the positive impact of GNI in maternal mortality reduction in Rwanda.

Though the forecasted maternal mortality in the next three years was not significantly increased, GNI showed a substantial impact to keep steadily downing mortalities.

## 5. Limitations

This study lacked access to strategical subjected causes of maternal mortalities on either pregnant women or the view of midwives, and primary data. This disturbs the contribution of external factors in model fitting. However, future research can be addressed in the investigation of the main cause's effect and related complications, which leads to maternal mortalities, especially in rural villages.

## 6. Conclusion

Despite steady forecasted maternal mortality within the next three years, the impacts of GNI to reduce these mortalities are not proportional to the needed targets of both MDGs and SDGs. Regardless of the complication of DESM, it is the best model to produce reliable forecasts, while the linear regression and ARIMA models can be used for quick forecasting. It is recommended that the Government of Rwanda shall use a substantial share of GNI in the health-care system, encourage regular prenatal consultation, and provide regular professional training to midwives and devolution of infrastructure to support pregnant women to reduce maternal mortality in advanced level.

## Figures and Tables

**Table 1:** Model parameters and the relationship between MMs and GNI.

Monthly mortality				Yearly mortality				Pearson correlation	
Lags	ACF	PACF	Q-stat	*P* value	ACF	PACF	Q-stat	*P*-value	MMs and GNI	*P* value

1	0.850	0.850	13.87	0.00	0.591	0.591	6.7129	0.010	−0.610	0.012
2	0.660	−0.22	22.84	0.00	0.268	−0.125	8.1908			
3	0.460	−0.13	27.53	0.00	0.283	0.279	9.9636	0.019		
4	0.266	−0.10	29.23	0.00	0.124	−0.263	10.335	0.035		
Model information	Parameters	Estimates	SE					*P* value		

DESM	Alpha (Level)	0.50	.019					.958		
	Gamma	0.201	0.201					.962		
	Beta	0.100								

ARIMA (0 1 0)	drift	3.39	0.23					0.000		
	alpha	1	0.00					0.003		
Linear model	Intercept alpha	3.32	0.036					0.114		
R-square (0.56)	alpha	0.011								
Nonparameter test	Stat-value	Bera-Jarque	Test-value							
Mann–Whitney U	46.500	Skewness	1.195							
Wilcoxon W	101.500	Kurtosis	2.329							
Z-test	−1.299	*P* value (2-tailed)	0.194							

ACF: autocorrelation function, PACF: partial autocorrelation function, MM: maternal mortality, GNI: gross national income, SE : standard error, AIC: Akaike information criteria. *P* value < 0.01 significant level.

**Table 2:** Models fitting.

Model	Fitted model
DSEM	(i) *S*_*t*_=0.001 *Y*_*t*_+(1 − 0.001)(*S*_*t*−1_+*b*_*t*−1_) *b*_*t*_=0.305(*S*_*t*_ − *S*_*t*−1_)+(1 − 0.305)*b*_*t*−1_
ARIMA	(ii) $\hat{{\text{Y}}_{\text{t}}}=3.93+{\text{Y}}_{\text{t}-1}$
Linear regression	(iii) *Y*_*t*_=3.312+0.011*t*+*ε*_*t*_

**Table 3:** Forecasted maternal mortality using different models.

Time	ARIMA (0, 1, O)		Linear model	DESM	
May 2018	6.93 ± 2.28		4.533 ± 2.34	4 [0 8]	
June 2018	9.93 ± 2.37		4.544 ± 2.47	4 [0 8]	
July 2018	12.93 ± 2.29		4.555 ± 2.26	6 [2 10]	
August 2018	15.93 ± 2.47		4.566 ± 2.37	6 [2 10]	
	Predicted value	Actual value	Forecast value	Monthly limits	Monthly errors
5/2016–5/2017	50	47		[−1, 10]	[−3, 3]
6/2017–5/2018	52	54		[−1, 11]	[−3, 7]
6/2018–5/2019			51	[0, 10]	[−3, 7]
6/2019–5/2020			48	[0, 10]	[−2, 9]
6/2020–5/2021			48	[0, 10]	[−1, 7]

## Data Availability

All computation dataset is available from the corresponding author and can be accessed on specific reason.

## Abbreviations

GNI: Gross national income
WHO: World Health Organization
DESM: Double exponential smoothing model
CHUK: Central Hospital of the University of Kigali
MDGs: Millennium development goals
SDGs: Sustainable development goals
ARIMA: Autoregressive integrated moving average.

## Supplementary Materials

Supplementary Materials Additional data file contains the most important computations of model parameters, validation criteria, and correlations between GNI and yearly and monthly maternal mortality by using some types of software.
