# Supplementary material for: Forecasting Maternal Complications Based on the Impact of Gross National Income Using Various Models for Rwanda
Source: J Environ Public Health. 2020 Aug 19;2020:7692428. doi: 10.1155/2020/7692428 (PMC7453229; doi:10.1155/2020/7692428)
Supplement: Supplementary Materials — Additional data file contains the most important computations of model parameters, validation criteria, and correlations between GNI and yearly and monthly maternal mortality by using some types of software. [file 7692428.f1.zip › 7692428.f1/Supplementary Materials/Additional file.docx]

| **Computations**  Date: 08/21/19 Time: 11:00 | | | |  |  |  |
| --- | --- | --- | --- | --- | --- | --- |
| Sample: 2002 2017 | |  |  |  |  |  |
| Included observations: 16 | | |  |  |  |  |
|  |  |  |  |  |  |  |
|  |  |  |  |  |  |  |
| Autocorrelation | Partial Correlation |  | AC | PAC | Q-Stat | Prob |
|  |  |  |  |  |  |  |
|  |  |  |  |  |  |  |
| . \|******\| | . \|******\| | 1 | 0.850 | 0.850 | 13.879 | 0.000 |
| . \|***** \| | . **\| . \| | 2 | 0.660 | -0.227 | 22.840 | 0.000 |
| . \|***. \| | . *\| . \| | 3 | 0.460 | -0.135 | 27.531 | 0.000 |
| . \|** . \| | . *\| . \| | 4 | 0.266 | -0.108 | 29.235 | 0.000 |

| YEARLY |  |  |  | | |
| --- | --- | --- | --- | --- | --- |
| \| Date: 08/21/19 Time: 15:40 \| \| \| \|  \|  \|  \| \| --- \| --- \| --- \| --- \| --- \| --- \| --- \| \| Sample: 2002 2017 \| \|  \|  \|  \|  \|  \| \| Included observations: 16 \| \| \|  \|  \|  \|  \| \|  \|  \|  \|  \|  \|  \|  \| \|  \|  \|  \|  \|  \|  \|  \| \| Autocorrelation \| Partial Correlation \|  \| AC \| PAC \| Q-Stat \| Prob \| \|  \|  \|  \|  \|  \|  \|  \| \|  \|  \|  \|  \|  \|  \|  \| \| . \|**** \| \| . \|**** \| \| 1 \| 0.591 \| 0.591 \| 6.7129 \| 0.010 \| \| . \|** . \| \| . *\| . \| \| 2 \| 0.268 \| -0.125 \| 8.1908 \| 0.017 \| \| . \|** . \| \| . \|** . \| \| 3 \| 0.283 \| 0.279 \| 9.9636 \| 0.019 \| \| . \|* . \| \| . **\| . \| \| 4 \| 0.124 \| -0.263 \| 10.335 \| 0.035 \| | \| Date: 08/21/19 Time: 15:40 \| \| \| \|  \|  \|  \| \| --- \| --- \| --- \| --- \| --- \| --- \| --- \| \| Sample: 2002 2017 \| \|  \|  \|  \|  \|  \| \| Included observations: 16 \| \| \|  \|  \|  \|  \| \|  \|  \|  \|  \|  \|  \|  \| \|  \|  \|  \|  \|  \|  \|  \| \| Autocorrelation \| Partial Correlation \|  \| AC \| PAC \| Q-Stat \| Prob \| \|  \|  \|  \|  \|  \|  \|  \| \|  \|  \|  \|  \|  \|  \|  \| \| . \|**** \| \| . \|**** \| \| 1 \| 0.591 \| 0.591 \| 6.7129 \| 0.010 \| \| . \|** . \| \| . *\| . \| \| 2 \| 0.268 \| -0.125 \| 8.1908 \| 0.017 \| \| . \|** . \| \| . \|** . \| \| 3 \| 0.283 \| 0.279 \| 9.9636 \| 0.019 \| \| . \|* . \| \| . **\| . \| \| 4 \| 0.124 \| -0.263 \| 10.335 \| 0.035 \| | \| Date: 08/21/19 Time: 15:40 \| \| \| \|  \|  \|  \| \| --- \| --- \| --- \| --- \| --- \| --- \| --- \| \| Sample: 2002 2017 \| \|  \|  \|  \|  \|  \| \| Included observations: 16 \| \| \|  \|  \|  \|  \| \|  \|  \|  \|  \|  \|  \|  \| \|  \|  \|  \|  \|  \|  \|  \| \| Autocorrelation \| Partial Correlation \|  \| AC \| PAC \| Q-Stat \| Prob \| \|  \|  \|  \|  \|  \|  \|  \| \|  \|  \|  \|  \|  \|  \|  \| \| . \|**** \| \| . \|**** \| \| 1 \| 0.591 \| 0.591 \| 6.7129 \| 0.010 \| \| . \|** . \| \| . *\| . \| \| 2 \| 0.268 \| -0.125 \| 8.1908 \| 0.017 \| \| . \|** . \| \| . \|** . \| \| 3 \| 0.283 \| 0.279 \| 9.9636 \| 0.019 \| \| . \|* . \| \| . **\| . \| \| 4 \| 0.124 \| -0.263 \| 10.335 \| 0.035 \| | \| Date: 08/21/19 Time: 15:40 \| \| \| \|  \|  \|  \| \| --- \| --- \| --- \| --- \| --- \| --- \| --- \| \| Sample: 2002 2017 \| \|  \|  \|  \|  \|  \| \| Included observations: 16 \| \| \|  \|  \|  \|  \| \|  \|  \|  \|  \|  \|  \|  \| \|  \|  \|  \|  \|  \|  \|  \| \| Autocorrelation \| Partial Correlation \|  \| AC \| PAC \| Q-Stat \| Prob \| \|  \|  \|  \|  \|  \|  \|  \| \|  \|  \|  \|  \|  \|  \|  \| \| . \|**** \| \| . \|**** \| \| 1 \| 0.591 \| 0.591 \| 6.7129 \| 0.010 \| \| . \|** . \| \| . *\| . \| \| 2 \| 0.268 \| -0.125 \| 8.1908 \| 0.017 \| \| . \|** . \| \| . \|** . \| \| 3 \| 0.283 \| 0.279 \| 9.9636 \| 0.019 \| \| . \|* . \| \| . **\| . \| \| 4 \| 0.124 \| -0.263 \| 10.335 \| 0.035 \| | | |
| ETS Smoothing | | | | |  |
| Original series: MATERNAL_MORTALITY | | | | |  |
| Date: 08/21/19 Time: 10:21 | | | | |  |
| Sample: 2009M01 2018M02 | | | | |  |
| Included observations: 110 | | | | |  |
| Model: A,AD,A - Additive Error, Additive-Dampened | | | | |  |
| Trend, Additive Season | | | | |  |
| Failure to improve objective (non-zero gradients) after 0 iterations | | | | |  |
|  | | | |  |  |
|  | | | |  |  |
| Parameters | | | | |  |
|  | | | |  |  |
|  | | | |  |  |
| Alpha: | | | | 0.500000 |  |
| Beta: | | | | 0.100080 |  |
| Gamma: | | | | 0.201960 |  |
| Phi: | | | | 0.978200 |  |
|  | | | |  |  |
|  | | | |  |  |
| Initial Parameters | | | | |  |
|  | | | |  |  |
|  | | | |  |  |
| Initial level: | | | | 4.267135 |  |
| Initial trend: | | | | -0.004704 |  |
| Initial state 1: | | | | -0.136863 |  |
| Initial state 2: | | | | -1.496238 |  |
| Initial state 3: | | | | 0.055845 |  |
| Initial state 4: | | | | 0.847512 |  |
| Initial state 5: | | | | 0.413484 |  |
| Initial state 6: | | | | -0.044850 |  |
| Initial state 7: | | | | -1.428530 |  |
| Initial state 8: | | | | -0.798322 |  |
| Initial state 9: | | | | -0.147280 |  |
| Initial state 10: | | | | 1.821470 |  |
| Initial state 11: | | | | 0.149595 |  |
| Initial state 12: | | | | 0.764178 |  |
|  | | | |  |  |
|  | | | |  |  |
| Compact Log-likelihood | | | | -385.8385 |  |
| Log-likelihood | | | | -283.3953 |  |
| Akaike Information Criterion | | | | 805.6770 |  |
| Schwarz Criterion | | | | 851.5852 |  |
| Hannan-Quinn Criterion | | | | 824.2976 |  |
| Sum of Squared Residuals | | | | 1113.480 |  |
| Root Mean Squared Error | | | | 3.181595 |  |

|  |  |  |  |  |
| --- | --- | --- | --- | --- |
|  |  |  |  |  |
|  | AC | PAC | Q-Stat | Prob |
|  |  |  |  |  |
|  |  |  |  |  |
| 1 | 0.850 | 0.850 | 13.879 | 0.000 |
| 2 | 0.660 | -0.227 | 22.840 | 0.000 |
| 3 | 0.460 | -0.135 | 27.531 | 0.000 |
| 4 | 0.266 | -0.108 | 29.235 | 0.000 |
|  |  |  |  |  |

**Mortality plots**


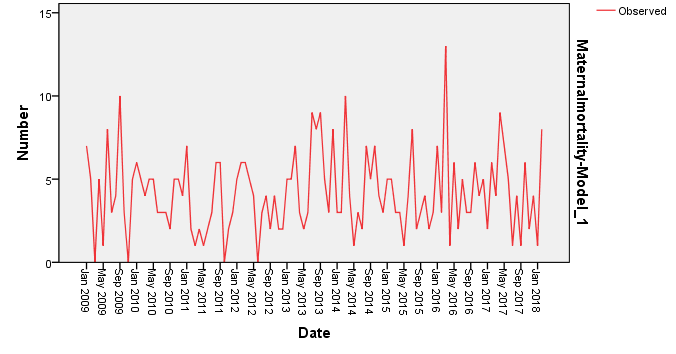


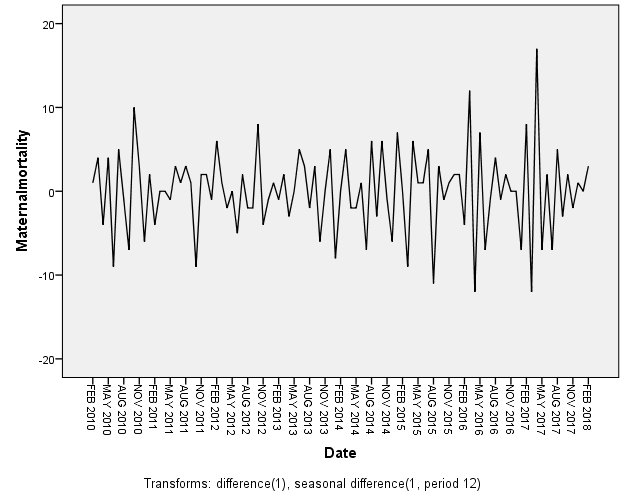


| **Correlations** | | | | |
| --- | --- | --- | --- | --- |
|  | | YEARLY | MORTALITY | GNI |
| YEARLY | Pearson Correlation | 1 | -.610^*^ | .944^**^ |
|  | Sig. (2-tailed) |  | .012 | .000 |
|  | Sum of Squares and Cross-products | 340.000 | -605.000 | 16510.000 |
|  | Covariance | 22.667 | -40.333 | 1100.667 |
|  | N | 16 | 16 | 16 |
| MORTALITY | Pearson Correlation | -.610^*^ | 1 | -.778^**^ |
|  | Sig. (2-tailed) | .012 |  | .000 |
|  | Sum of Squares and Cross-products | -605.000 | 2891.000 | -39695.000 |
|  | Covariance | -40.333 | 192.733 | -2646.333 |
|  | N | 16 | 16 | 16 |
| GNI | Pearson Correlation | .944^**^ | -.778^**^ | 1 |
|  | Sig. (2-tailed) | .000 | .000 |  |
|  | Sum of Squares and Cross-products | 16510.000 | -39695.000 | 899575.000 |
|  | Covariance | 1100.667 | -2646.333 | 59971.667 |
|  | N | 16 | 16 | 16 |
| *. Correlation is significant at the 0.05 level (2-tailed). | | | | |
| **. Correlation is significant at the 0.01 level (2-tailed). | | | | |
