# Supplementary material for: Forecasting Maternal Complications Based on the Impact of Gross National Income Using Various Models for Rwanda
Source: J Environ Public Health. 2020 Aug 19;2020:7692428. doi: 10.1155/2020/7692428 (PMC7453229; doi:10.1155/2020/7692428)
Supplement: Supplementary Materials — Additional data file contains the most important computations of model parameters, validation criteria, and correlations between GNI and yearly and monthly maternal mortality by using some types of software. [file 7692428.f1.zip › 7692428.f1/Supplementary Materials/data-11.docx]

Table 1. Data used in the study

| YEARLY | MORTALITY | GNI |
| --- | --- | --- |
| 2002 | 85 | 720 |
| 2003 | 80 | 730 |
| 2004 | 73 | 790 |
| 2005 | 67 | 880 |
| 2006 | 62 | 960 |
| 2007 | 57 | 1030 |
| 2008 | 53 | 1130 |
| 2009 | 49 | 1180 |
| 2010 | 49 | 1230 |
| 2011 | 35 | 1310 |
| 2012 | 41 | 1390 |
| 2013 | 67 | 1350 |
| 2014 | 52 | 1350 |
| 2015 | 43 | 1350 |
| 2016 | 58 | 1350 |
| 2017 | 61 | 1350 |

Table 2: Monthly maternal mortality

| Time | Maternal mortality |
| --- | --- |
| 2009/1 | 7 |
| 2009/2 | 5 |
| 2009/3 | 0 |
| 2009/4 | 5 |
| 2009/5 | 1 |
| 2009/6 | 8 |
| 2009/7 | 3 |
| 2009/8 | 4 |
| 2009/9 | 10 |
| 2009/10 | 3 |
| 2009/11 | 0 |
| 2009/12 | 5 |
| 2010/1 | 6 |
| 2010/2 | 5 |
| 2010/3 | 4 |
| 2010/4 | 5 |
| 2010/5 | 5 |
| 2010/6 | 3 |
| 2010/7 | 3 |
| 2010/8 | 3 |
| 2010/9 | 2 |
| 2010/10 | 5 |
| 2010/11 | 5 |
| 2010/12 | 4 |
| 2011/1 | 7 |
| 2011/2 | 2 |
| 2011/3 | 1 |
| 2011/4 | 2 |
| 2011/5 | 1 |
| 2011/6 | 2 |
| 2011/7 | 3 |
| 2011/8 | 6 |
| 2011/9 | 6 |
| 2011/10 | 0 |
| 2011/11 | 2 |
| 2011/12 | 3 |
| 2012/1 | 5 |
| 2012/2 | 6 |
| 2012/3 | 6 |
| 2012/4 | 5 |
| 2012/5 | 4 |
| 2012/6 | 0 |
| 2012/7 | 3 |
| 2012/8 | 4 |
| 2012/9 | 2 |
| 2012/10 | 4 |
| 2012/11 | 2 |
| 2012/12 | 2 |
| 2013/1 | 5 |
| 2013/2 | 5 |
| 2013/3 | 7 |
| 2013/4 | 3 |
| 2013/5 | 2 |
| 2013/6 | 3 |
| 2013/7 | 9 |
| 2013/8 | 8 |
| 2013/9 | 9 |
| 2013/10 | 5 |
| 2013/11 | 3 |
| 2013/12 | 8 |
| 2014/1 | 3 |
| 2014/2 | 3 |
| 2014/3 | 10 |
| 2014/4 | 4 |
| 2014/5 | 1 |
| 2014/6 | 3 |
| 2014/7 | 2 |
| 2014/8 | 7 |
| 2014/9 | 5 |
| 2014/10 | 7 |
| 2014/11 | 4 |
| 2014/12 | 3 |
| 2015/1 | 5 |
| 2015/2 | 5 |
| 2015/3 | 3 |
| 2015/4 | 3 |
| 2015/5 | 1 |
| 2015/6 | 4 |
| 2015/7 | 8 |
| 2015/8 | 2 |
| 2015/9 | 3 |
| 2015/10 | 4 |
| 2015/11 | 2 |
| 2015/12 | 3 |
| 2016/1 | 7 |
| 2016/2 | 3 |
| 2016/3 | 13 |
| 2016/4 | 1 |
| 2016/5 | 6 |
| 2016/6 | 2 |
| 2016/7 | 5 |
| 2016/8 | 3 |
| 2016/9 | 3 |
| 2016/10 | 6 |
| 2016/11 | 4 |
| 2016/12 | 5 |
| 2017/1 | 2 |
| 2017/2 | 6 |
| 2017/3 | 4 |
| 2017/4 | 9 |
| 2017/5 | 7 |
| 2017/6 | 5 |
| 2017/7 | 1 |
| 2017/8 | 4 |
| 2017/9 | 1 |
| 2017/10 | 6 |
| 2017/11 | 2 |
| 2017/12 | 4 |
| 2018/1 | 1 |
| 2018/2 | 8 |
